# Circulating choline levels are associated with prognoses in patients with pulmonary hypertension: a cohort study

**DOI:** 10.1186/s12890-023-02547-9

**Published:** 2023-09-10

**Authors:** Yicheng Yang, Beilan Yang, Bingyang Liu, Yanru Liang, Qin Luo, Zhihui Zhao, Zhihong Liu, Qixian Zeng, Changming Xiong

**Affiliations:** Center of Pulmonary Vascular Disease, State Key Laboratory of Cardiovascular Disease, Fuwai Hospital, National Center for Cardiovascular Disease, Chinese Academy of Medical Sciences, Peking Union Medical College, Beijing, 100037 China

**Keywords:** Pulmonary hypertension, Choline, Trimethylamine oxide, Metabolite, Prognoses

## Abstract

**Backgrounds:**

Mounting evidences have highlighted the association between metabolites and cardiovascular diseases. Our previous works have demonstrated that circulating metabolite, trimethylamine oxide, was associated with prognosis of patients with pulmonary hypertension (PH). Choline is a precursor of trimethylamine oxide and its role in PH remains unknown. Here, we aimed to validate the hypothesis that circulating choline levels were associated with prognoses in patients with PH.

**Methods:**

Inpatients diagnosed with PH—defined as mean pulmonary arterial pressure ≥ 25 mmHg by right heart catheterisation—from Fuwai Hospital were enrolled after excluding relative comorbidities. Fasting blood samples were obtained to assess choline levels and other clinical variables. The primary endpoints were defined as death, escalation of targeted medication, rehospitalization due to heart failure, PH deterioration. The follow-up duration was defined as the time from the choline examination to the occurrence of outcomes or the end of the study. The associations between circulating choline levels and disease severity and prognoses were explored.

**Results:**

Totally, 272 inpatients with PH were enrolled in this study. Patients were divided into high and low choline groups according to the 50^th^ quartile of circulating choline levels, defined as 12.6 µM. After confounders adjustment, the high circulating choline levels were still associated with poor World Health Organization functional class, elevated N-terminal pro-B-type natriuretic peptide, and decreased cardiac output index indicating the severe disease condition. Moreover, elevated choline levels were associated with poor prognoses in PH patients even after adjusting for confounders (hazard ratio = 1.934; 95% CI, 1.034–3.619; ***P*** = 0.039). Subgroup analyses showed that choline levels predicted the prognosis of patients with pulmonary arterial hypertension but not chronic thromboembolic pulmonary hypertension.

**Conclusions:**

Choline levels were associated with disease severity and poor prognoses of patients with PH, especially in pulmonary arterial hypertension suggesting its potential biomarker role.

**Supplementary Information:**

The online version contains supplementary material available at 10.1186/s12890-023-02547-9.

## Background

Pulmonary hypertension (PH) is an abnormal hemodynamic status characterised by increased vascular tone, progressive vascular remodelling, and elevated pulmonary vascular resistance. Although many advances have been made in the management of this disease, such as vasodilator drugs, PH remains an uncurable and fatal condition with an estimated 1-year mortality rate of > 10% among high-risk patients [1]. The grave prognoses and its intrinsic multi-aetiological nature indicate the multifactorial pathological mechanisms of PH, underscoring the need for a more comprehensive understanding of this devastating disease.

Mounting evidence has highlighted the association between metabolites and cardiovascular diseases [2, 3]. Trimethylamine oxide (TMAO), a gut microbiota-derived metabolite, promotes endothelial cell senescence and vascular aging by oxidative stress [4, 5]. There is increasing interest in the potential role of metabolism in the initiation and progression of PH as studies have suggested the presence of metabolic disturbances in patients with PH [6]. Our previous work also revealed that aberrant metabolism, characterised by elevated plasma TMAO and betaine levels, is associated with greater disease severity and worse prognoses in patients with PH and may serve as potential surrogate biomarkers for PH [7–9]. Choline—a precursor of TMAO—is a critical micronutrient that participates in various physiological processes, and it serves as an independent predictor of various cardiovascular disorders, such as hypertension [10], atherosclerosis [11], and myocardial infarction [12]. However, the relationship between choline and PH, a severe haemodynamic abnormality, remains largely unexplored.

Therefore, in the present study, we investigated the effect of baseline plasma choline levels on PH to evaluate its potential as a surrogate biomarker of PH.

## Methods

### Study design and patients

Clinical research was carried out according to The Code of Ethics of the World Medical Association (Declaration of Helsinki). Written informed consent was obtained, and the ethics committee of Fuwai Hospital approved the study.

This study included inpatients diagnosed with PH—defined as mean pulmonary arterial pressure ≥ 25 mmHg by right heart catheterisation—from April 2019 to March 2020 in the Pulmonary Vascular Diseases Centre of Fuwai Hospital. Patients with a history of gastrointestinal surgery, mental illness, acute heart failure, and liver diseases, including hepatitis, cirrhosis, and alcoholic and non-alcoholic fatty liver disease, were excluded.

Demographic characteristics, World Health Organization functional class (WHO-FC), fasting choline and biochemical indicators, 6-minute walk distance (6 MWD), and echocardiographic parameters were collected. Additionally, cardiopulmonary exercise tests and haemodynamic examinations were performed to further evaluate the disease condition.

### Study endpoint and follow-up

The primary endpoints were defined as death, escalation of targeted medication, rehospitalisation due to heart failure, PH deterioration including worsening symptoms, higher WHO-FC compared with baseline, or at least a 15% decrease in the 6 MWD [13]. The follow-up duration was defined as the time between the choline examination and occurrence of outcomes or study end. The median follow-up duration in this study was 1.1 (interquartile range: 0.6, 1.7) years.

### Quantification of choline

Fasting blood samples were obtained from all PH patients. After centrifugation for 10 min at 3,000 r/min, the supernatants were collected and stored at -80℃. The samples were ordered randomly and shipped on dry ice to the Institute of Cardiovascular Sciences and Institute of Systems Biomedicine (Peking University, Beijing, China) for metabolomics analyses. The detailed detection procedures were the same as those used in the quantification of metabolites shown in our previous study [5]. Three quality-control samples with different choline concentrations were measured every 20 samples.

### Statistical analysis

The linearity between choline and the primary endpoint was analysed using a restricted cubic spline. The patients with PH were divided into two cohorts according to the 50^th^ quartile of circulating choline levels. Student’s t-test or Wilcoxon rank-sum test for continuous data and Chi-square test for categorical variables were used to evaluate discrepancies among groups. Correlations between choline levels and clinical variables were explored using Spearman’s correlation (two-tailed) and generalised linear models. Kaplan–Meier (KM) analysis and Cox proportional hazards regression were used to further explore hazard ratios (HRs) and 95% confidence intervals (CIs) in the high-choline group. Statistical significance was defined as a two-sided *P* < 0.05. Analyses were performed using R 2.8.0 (Vienna, Austria) and SPSS (version 23; IBM Corp, 2015).

## Results

### Study population and baseline demonstration

**Fig. 1:**
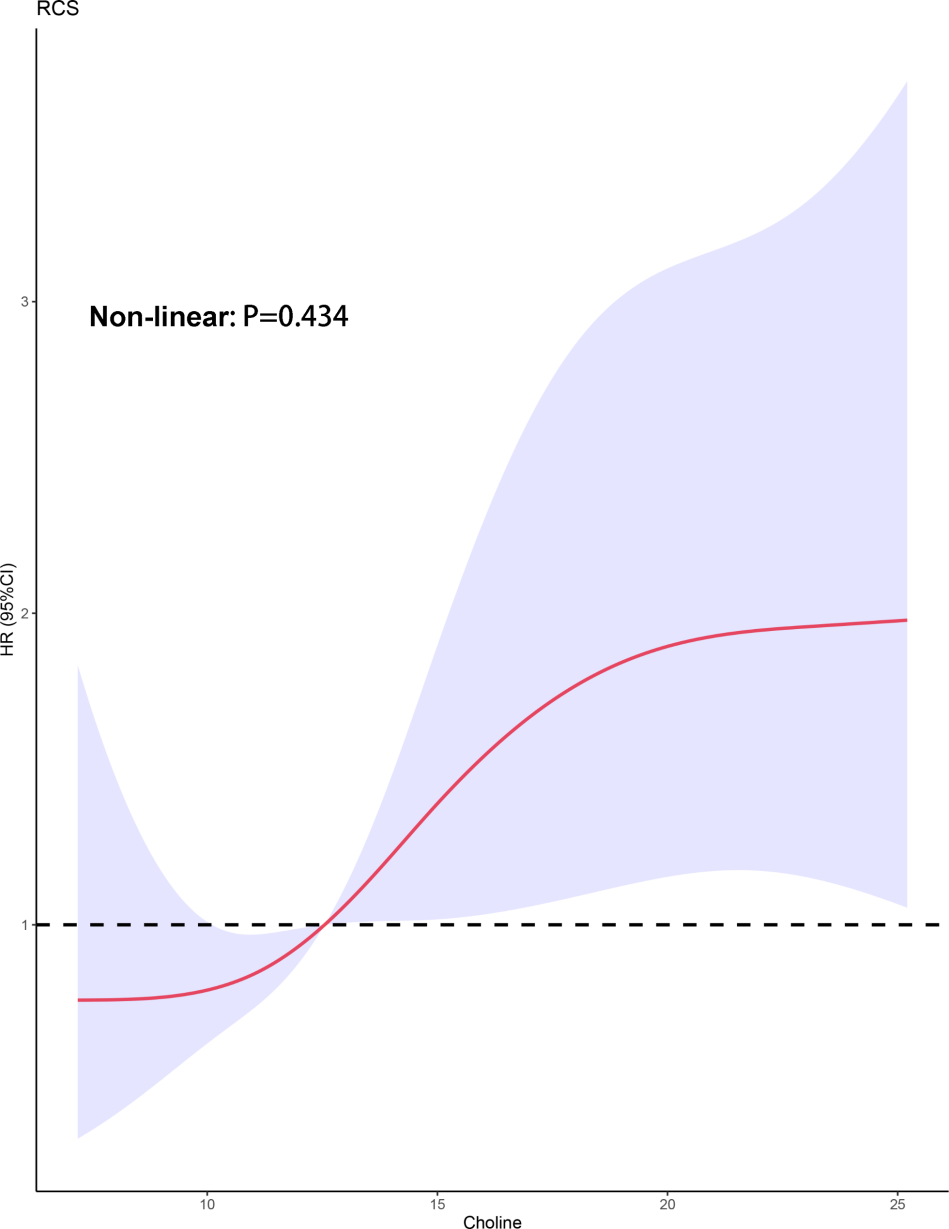
Restricted cubic spline result of choline levels in relation to hazard ratio for the risk of clinical outcomes (n = 272). Red line with 95% confidence interval shaded in light purple. HR: hazard ratio; CI: confidence interval

A total of 272 inpatients with PH, including 56 with idiopathic/heritable pulmonary arterial hypertension (IPAH/HPAH), 101 with PAH-associated with congenital heart disease (CHD-PAH), 78 with chronic thromboembolic pulmonary hypertension (CTEPH), and 37 with other PH types, were included in this cohort study. The linear correlation between choline levels and clinical outcomes assessed using a restricted cubic spline is shown in Fig. 1 (nonlinear, *P* = 0.434). The 50th quartile of circulating choline level was detected as 12.6 µM, and patients were divided into high and low choline groups according to the cut-off value. Table 1 shows the baseline characteristics of the two cohorts, indicating that patients with high choline levels exhibited a worse WHO-FC, greater decrease in exercise tolerance, and more severe haemodynamic status than those with low metabolite levels.

**Table 1:** Characteristics of total PH patients stratified by 50th percentile of plasma choline levels

Variables	Total PH patients N = 272	High Choline group N = 136	Low Choline group N = 136	*P* value
Age, years	43.0 ± 16.5	46.3 ± 17.0	39.7 ± 15.3	**0.001**
Female sex, n (%)	176 (64.7)	70 (51.5)	106 (77.9)	**< 0.001**
BMI, kg/m^2^	22.2 ± 4.6	22.5 ± 4.7	22.0 ± 4.5	0.414
**WHO-FC, n (%)**				
I-II	168 (61.8)	71 (52.2)	97 (71.3)	**0.001**
III-IV	104 (38.2)	65 (47.8)	39 (28.7)	**0.001**
**Laboratories**				
Choline, µM	12.6 (10.1, 16.7)	16.6 (14.5, 19.3)	10.1 (8.4, 11.3)	**< 0.001**
NT-proBNP, pg/ml	499.4 (150.1, 1565.3)	977.9 (232.9, 2402.8)	298.3 (137.6, 716.8)	**< 0.001**
ALT, IU/L	18.0 (11.3, 26.0)	19.0 (12.0, 30.0)	17.0 (11.0, 24.8)	0.122
AST, IU/L	27.0 (22.0, 34.0)	28.0 (23.0, 34.0)	26.0 (21.3, 34.0)	0.135
Creatinine, µM	77.0 (66.6, 91.0)	85.5 (72.0, 97.9)	71.0 (63.0, 80.7)	**< 0.001**
Total cholesterol, mM	4.1 ± 1.1	4.0 ± 1.1	4.2 ± 1.2	0.176
Triglycerides, mM	1.3 ± 0.9	1.3 ± 0.5	1.3 ± 1.2	0.657
Serum iron, µM	16.1 ± 7.9	15.9 ± 8.0	14.9 ± 7.5	0.271
**Exercise capacity**				
PeakVO_2_, mL/min/kg	14.1 ± 3.9	13.1 ± 3.8	15.1 ± 3.8	**< 0.001**
VO_2_%	1.5 ± 0.3	1.4 ± 0.3	1.6 ± 0.3	**< 0.001**
VCO_2_%	1.4 ± 0.3	1.3 ± 0.3	1.5 ± 0.3	**< 0.001**
6MWD, m	414.1 ± 99.4	400.1 ± 104.1	429.2 ± 91.5	**0.033**
**Hemodynamics**				
mRAP, mmHg	6.5 ± 4.1	6.6 ± 4.4	6.3 ± 3.7	0.580
RVDP, mmHg	-1.0 (-5.5, 3.0)	-0.5 (-5.0, 4.0)	-2.0 (-6.0, 2.0)	**0.020**
mPAP, mmHg	57.4 ± 17.4	58.1 ± 17.7	56.8 ± 17.2	0.588
Cardiac output index, L/min*m^2^	3.1 ± 1.0	2.9 ± 0.9	3.4 ± 1.0	**0.001**
**Targeted treatment**				
ERA	142 (52.2)	63 (46.3)	79 (58.1)	0.068
NO pathway	214 (78.7)	103 (75.7)	111 (81.6)	0.159
Prostacyclin analogues	43 (15.8)	25 (18.4)	18 (13.2)	0.150

PH patients were stratified into low and high choline groups by 50th percentile of plasma choline levels (12.6 µM). PH: pulmonary hypertension; BMI: body mass index; WHO FC: world health organization function class; NT-proBNP: N-terminal pro-brain natriuretic peptide; ALT: alanine aminotransferase; AST: aspartate aminotransferase; 6MWD: 6-minute walk distance; mRAP: mean right atrial pressure; RVDP: right ventricular diastolic pressure; mPAP: mean pulmonary atrial pressure; ERA: endothelium-Receptor Antagonist; NO: nitric oxide

### The association between choline levels and disease severity after confounder adjustment in the total cohort

The correlations between choline levels and clinical variables in all patients with PH are shown in Table S1. After adjusting for confounding clinical factors, a high circulating choline level remained associated with poor WHO-FC (odds ratio [OR] = 3.967; 95% CI, 1.444–10.901; *P* = 0.008), elevated N-terminal pro-B-type natriuretic peptide (NT-proBNP) levels (OR = 3.275; 95% CI, 1.322–8.110; *P* = 0.010), and decreased cardiac output index (β=-0.187; 95% CI, -0.706 to 0.027; *P* = 0.035). Moreover, this conclusion remained robust even after adjusting for comorbidities, including hypertension, coronary heart disease, and diabetes (Table S2).

### High choline levels predicted poor prognoses in patients with PH

**Fig. 2:**
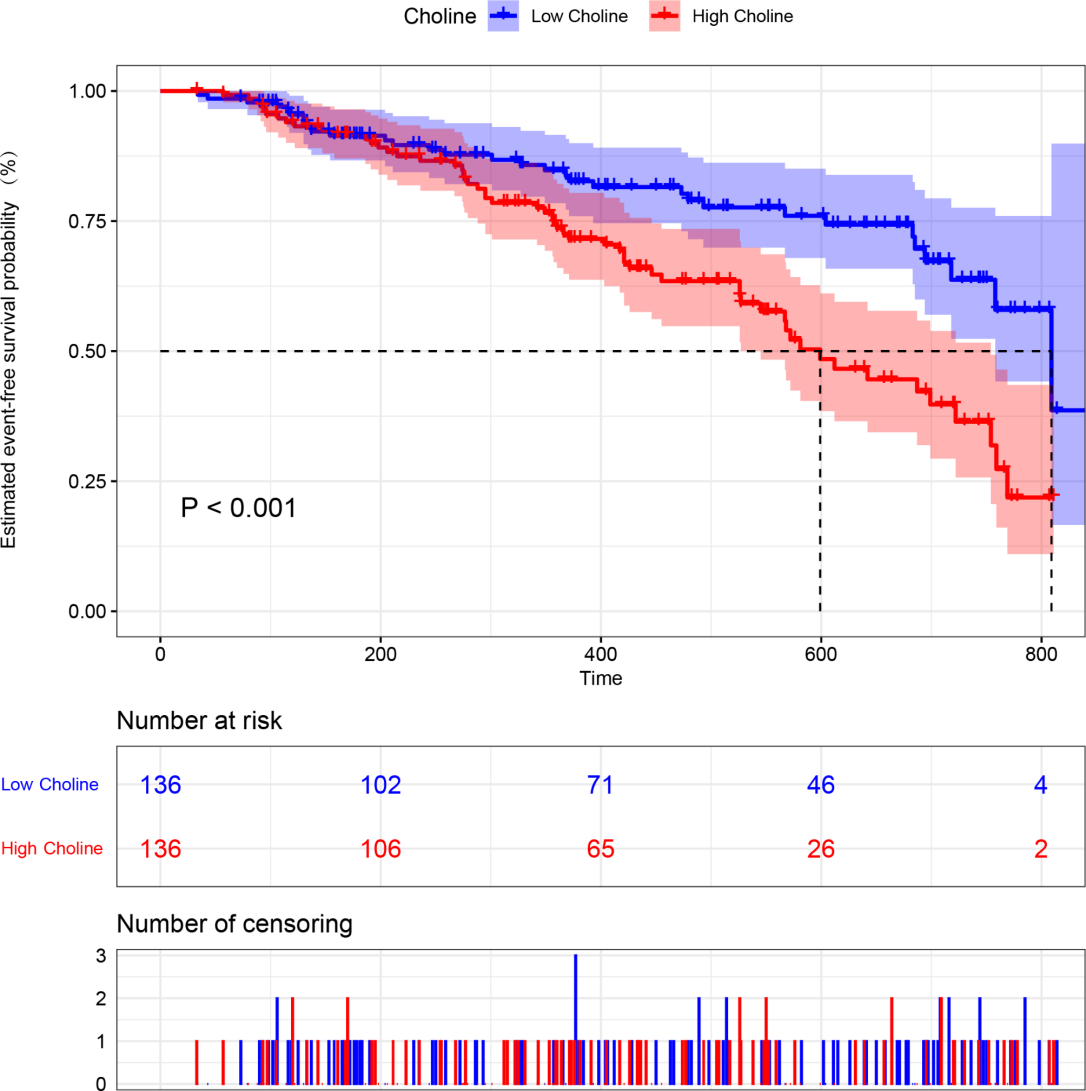
Kaplan–Meier analysis for the incidence of composite outcome events in all PH patients with high and low choline levels. Two hundred and seventy-two patients were analysed (n = 136 both in the high and low choline group). *P*-value calculated using the log-rank test. PH: pulmonary hypertension

Patients with PH and high circulating choline levels had poorer prognoses than did those with low levels (*P* < 0.001; Fig. 2). In accordance with the univariate Cox regression analysis (Table S3), confounding factors, including age, NT-proBNP, Creatinine, peak VO_2_, and 6 MWD, were adjusted, and the results demonstrated that a high choline level was still associated with poor prognoses in the total PH cohort (HR = 1.934; 95% CI, 1.034–3.619; *P* = 0.039; Table 2).

**Table 2:** Multivariate Cox analysis of choline and clinical outcomes in total PH patients

Variable	HR	95% CI	*P*
Choline	1.934	1.034–3.619	0.039
Age, years	1.003	0.984–1.021	0.781
NT-proBNP, pg/mL (categorical variable)	0.755	0.421–1.353	0.345
Creatinine, µM	1.002	1.000-1.005	0.035
PeakVO_2_, mL/min/kg	0.880	0.805–0.962	0.008
6MWD, m	1.001	0.421–1.353	0.724

Plasma choline levels were put into the model as a categorical variable bounded by 50th percentile (12.6 µM). PH: pulmonary hypertension; NT-proBNP: N-terminal pro-brain natriuretic peptide; 6MWD: 6-minute walk distance

### Subgroup analysis of choline and PH with different aetiologies

**Fig. 3:**
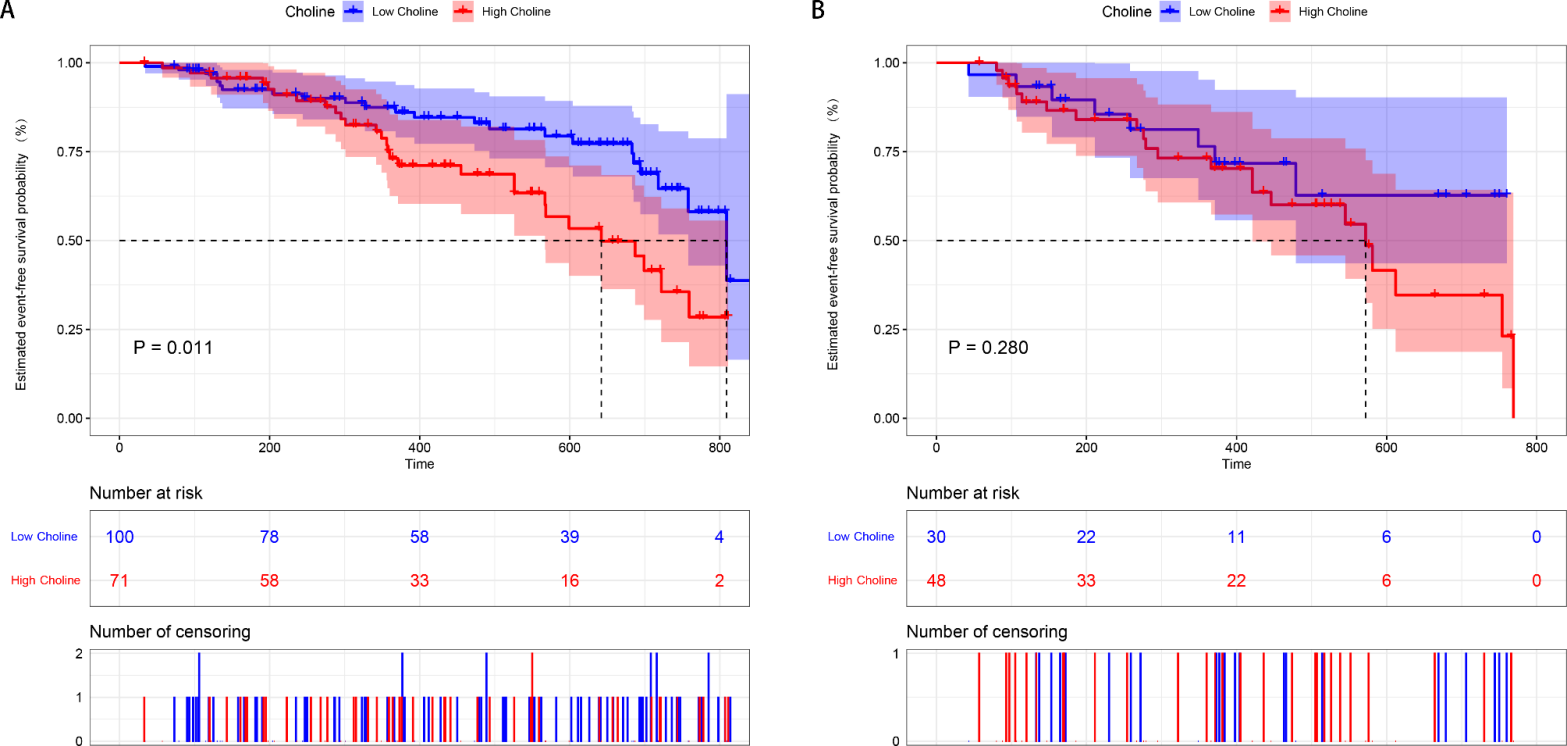
Kaplan–Meier analysis for the incidence of composite outcome events in PAH **(A)** and CTEPH subgroups **(B)**. One hundred and seventy-one patients with PAH and seventy-eight with CTEPH were enrolled for analysis. *P*-value calculated using the log-rank test. PAH: pulmonary arterial hypertension; CTEPH: chronic thromboembolic pulmonary hypertension

This section classifies patients with PH into IPAH/HPAH, CHD-PAH, and CTEPH for subgroup analysis. The baseline characteristics of different subgroups stratified by circulating choline levels are shown in Table S4, and similar trends between high choline levels and worse disease severity were elucidated. Survival analysis revealed that high choline levels predicted a poor prognosis among patients with PAH (including IPAH/HPAH and CHD-PAH, *P* = 0.011; Fig. 3A). Results of univariate Cox analysis between choline and clinical indicators in the PAH subgroup is shown in Table S5. Moreover, the proportional hazards assumption was examined using Schoenfeld residuals, and a violation of the model assumption was detected. Therefore, a time-dependent covariate was introduced. The results suggested that a high choline level still correlated with adverse outcomes in patients with PAH 8 months after adjusting for confounding factors (HR = 2.395; 95% CI, 1.072–5.352; *P* = 0.033; Table 3). However, no significant correlation was observed in the CTEPH subgroup (KM analysis, *P* = 0.280; Fig. 3B).

**Table 3:** Multivariate Cox analysis of choline and clinical outcomes in PAH subgroup

Variable	HR	95% CI	*P*
Choline * (time < 240 days)	0.894	0.194–4.114	0.886
Choline * (time **≥** **240 days)**	**2.395**	**1.072–5.352**	**0.033**
Age, years	0.985	0.959–1.012	0.262
BMI, kg/m^2^	0.969	0.891–1.055	0.468
WHO-FC	1.516	0.717–3.207	0.277
Total cholesterol, mM	1.113	0.768–1.613	0.573
Triglycerides, mM	1.056	0.649–1.717	0.828
6MWD, m	0.998	0.994–1.002	0.286

Plasma choline levels were put into the model as a categorical variable bounded by 50th percentile (12.6 µM). PAH: pulmonary arterial hypertension; BMI: body mass index; WHO FC: world health organization function class; 6MWT: 6-minute walk distance

## Discussion

The present study revealed that plasma choline levels were significantly correlated with several canonical risk indicators of PH. Patients with high circulating choline levels (above the 50th percentile of the study participants) had poorer prognoses. Patients with high choline levels were distinguished by older age, decreased cardiac output index, worsened WHO-FC and exercise capacity, and elevated NT-proBNP levels, right ventricular diastolic pressure, and creatinine levels. After adjusting for confounders, the associations between choline levels and disease severity remained robust [14]. Moreover, we found that high plasma choline levels were associated with poor prognoses. Patients with PH in the upper half of choline levels had an approximately 1.9-fold greater risk of adverse outcomes than those with lower choline levels.

In our study, choline levels were positively correlated with age and negatively correlated with female sex, consistent with the findings of a large population-based study in Norway [15]. This might be attributed to the distinct dietary patterns among patients of different ages, and oestrogen might result in a discrepancy in the choline levels between men and women [16]. According to previous studies, both the liver and kidney are involved in choline metabolism [17, 18], whereas in our study, no liver and kidney dysfunctions were observed in the high and low choline groups. Notably, although the creatinine levels in patients with elevated choline levels were higher than those in patients with low choline levels, the levels were still within the normal range, suggesting unimpaired kidney function. These results suggest that the discrepancy in choline levels among the patients cannot be simply attributed to the differences in liver and kidney function. Various tissues may be involved in the modulation of plasma choline levels, except for the organs mentioned above [17]. The WHO-FC, NT-proBNP, and cardiac output index are commonly used to evaluate PH severity and play essential roles in disease management [19]. After adjustment for confounders, high plasma choline levels were associated with severe disease condition as assessed by elevated NT-proBNP, worsened WHO-FC, and decreased cardiac output index in the two PH cohorts who received similar targeted medication. In line with those in all PH patients, the subgroup analysis results for different aetiologies also indicated significantly greater disease severity among patients with elevated baseline choline levels. Similarly, no interference from medical treatments was observed.

Additionally, we observed a strong inverse correlation between high choline levels and decreased exercise capacity. Taking up approximately 40% of the body mass, the skeletal muscle is one of the main reservoirs of choline in mammals [20], and it absorbs choline from the blood during exercise and releases it in the resting state [21]. Many diseases are complicated by muscular atrophy and hypofunction, including PH [22], heart failure [23], and chronic obstructive pulmonary disease [24]. Considering the debilitating aspects of PH, it is reasonable to presume that plasma choline levels in patients with PH might increase due to physical inactivity and muscle atrophy, especially in patients with advanced PH, resulting in disturbed plasma choline modulation.

Furthermore, we showed that patients with PH and high choline levels have a poor prognosis. Survival analysis in the subgroup revealed an independent association between baseline choline levels and the prognosis of patients with PAH 8 months later. In contrast, no differences were found in the survival outcomes of CTEPH patients with different choline levels according to the KM analysis. This result may be explained by the widely performed balloon pulmonary angioplasty during follow-up to treat CTEPH that drastically improves overall patient condition, leading to a diminished difference in the outcomes of patients with different baseline choline levels.

Whether elevated choline levels are a cause or effect in patients with PH with poor prognosis remains a question that needs to be discussed. On one hand, choline elevating passively in patients with worse conditions may serve as a compensatory response to counter the abnormal pathophysiological alterations in PH in a manner similar to that of NT-proBNP in heart failure. However, unlike endogenously secreted NT-proBNP, choline accumulation in the plasma may be derived from the unaffected or even enhanced hydrolysis of choline-containing esters and additive effect of reduced choline metabolism by cholinesterase. The uptake and transformation of choline in tissues depends on energy consumption. Under hypoxic or ischemic conditions, choline metabolism is reduced, resulting in increased choline levels [25, 26]. Additionally, the energy-independent hydrolysis of choline-containing esters enables persistently stable choline production, further accelerating choline accumulation [27]. Based on this knowledge, disturbances in choline production and metabolism might be attributed to the increase in plasma choline among patients with severe PH and right-heart failure who experienced systemic circulation congestion and hypoxia.

Conversely, circulating choline might contribute to PH development. TMAO, a gut microbiota-related metabolite of choline, is arguably deleterious [28]. The increase in TMAO levels may be responsible for the detrimental effects thought to originate directly from choline [29, 30]. We have demonstrated that TMAO levels are associated with poor prognoses in patients with PH [9] and that pulmonary vascular remodelling in monocrotaline-induced PH rats was ameliorated after TMAO decreased, followed by the regulation of proliferation, apoptosis, and vasodilator pathways [8]. Additionally, TMAO has been reported to aggregate PH by inducing macrophage/monocyte chemokines and cytokine production [31]. Whether choline promotes the progression of PH through the dysfunction of the above pathogeneses induced by TMAO elevation is worth exploring in future studies. Furthermore, the adverse effects of choline in cardiovascular disease have been elucidated. It has been reported dietary supplementation of choline in mice upregulated macrophage scavenger receptors, resulting in the promotion of atherosclerosis [32]. Specific choline pathogenesis independent of the TMAO pathway in PH and other cardiovascular diseases requires further exploration. Moreover, choline is a methyl donor involved in transmethylation pathways, including the synthesis of homocysteine, which is a cardiovascular disease risk factor. Homocysteine has also been recognized as a potential biomarker in PH [33, 34]. Choline may increase the homocysteine levels to promote PH development. Overall, the role of metabolites in the occurrence and development of diseases is complex, and multifunctional pathways are involved in this process. Further research is required to elucidate the underlying links and molecular mechanisms of choline in PH.

The single-centre design was the main limitation of this study. However, to the best of our knowledge, this is the first and largest study exploring the effect of baseline plasma choline levels on the severity and outcomes of patients with PH. Cholinesterase activity may influence choline plasma levels, and it is an interesting research direction to explore the association between cholinesterase and PH. Furthermore, patient dietary history was not obtained, and we could not rule out the impact of diet on choline levels among these patients. Measuring the choline levels several times during a certain period before grouping is recommended for future studies.

## Conclusions

Choline is associated with worsened severity and poor prognoses in patients with PH, particularly those with PAH indicating its potential biomarker role in PH.

### Electronic supplementary material

Below is the link to the electronic supplementary material.

Supplementary Material 1

Supplementary Material 2

Supplementary Material 3

Supplementary Material 4

Supplementary Material 5

## Data Availability

The datasets analysed during the current study are not publicly available due [REASON WHY DATA ARE NOT PUBLIC] but are available from the corresponding author on reasonable request.

## Abbreviations

PH: Pulmonary hypertension
TMAO: Trimethylamine oxide
WHO-FC: World Health Organization functional class (WHO-FC)
MWD: Minute walk distance (MWD)
KM: Kaplan-Meier (analysis)
HR: Hazard ratio
OR: Odds ratio
CI: Confidence interval
IPAH/HPAH: Idiopathic/heritable pulmonary arterial hypertension
CHD-PAH: PAH-associated with congenital heart disease
CTEPH: Chronic thromboembolic pulmonary hypertension
NT-proBNP: N-terminal pro-B-type natriuretic peptide levels

## References

[1] Humbert M, Kovacs G, Hoeper MM, Badagliacca R, Berger RMF, Brida M, Carlsen J, Coats AJS, Escribano-Subias P, Ferrari P et al. : 2022 ESC/ERS guidelines for the diagnosis and treatment of pulmonary hypertension. Eur Heart J 2022. 10.1183/13993003.00879-2022 36028254

[2] Ingwall JS (2009). Energy metabolism in heart failure and remodelling. Cardiovascular Res.

[3] Robaczewska J, Kedziora-Kornatowska K, Kozakiewicz M, Zary-Sikorska E, Pawluk H, Pawliszak W, Kedziora J (2016). Role of glutathione metabolism and glutathione-related antioxidant defense systems in hypertension. J Physiol pharmacology: official J Pol Physiological Soc.

[4] Ke Y, Li D, Zhao M, Liu C, Liu J, Zeng A, Shi X, Cheng S, Pan B, Zheng L (2018). Gut flora-dependent metabolite Trimethylamine-N-oxide accelerates endothelial cell senescence and vascular aging through oxidative stress. Free Radic Biol Med.

[5] Li D, Ke Y, Zhan R, Liu C, Zhao M, Zeng A, Shi X, Ji L, Cheng S, Pan B (2018). Trimethylamine-N-oxide promotes brain aging and cognitive impairment in mice. Aging Cell.

[6] Xu W, Janocha AJ, Erzurum SC (2021). Metabolism in Pulmonary Hypertension. Annu Rev Physiol.

[7] Yang Y, Xu J, Zhou J, Xue J, Gao J, Li X, Sun B, Yang B, Liu Z, Zhao Z (2022). High betaine and dynamic increase of Betaine levels are both Associated with Poor Prognosis of Patients with Pulmonary Hypertension. Front Cardiovasc Med.

[8] Yang Y, Zeng Q, Gao J, Yang B, Zhou J, Li K, Li L, Wang A, Li X, Liu Z et al. High circulating gut microbiota-dependent metabolite trimethylamine N-oxide is associated with poor prognosis in pulmonary arterial hypertension. Eur Heart J Open 2022. 10.1093/ehjopen/oeac021 PMC9442843 36071697

[9] Yang Y, Yang B, Li X, Xue L, Liu B, Liang Y, Zhao Z, Luo Q, Liu Z, Zeng Q (2022). Higher circulating trimethylamine N-oxide levels are associated with worse severity and prognosis in pulmonary hypertension: a cohort study. Respir Res.

[10] Song M, Xu BP, Liang Q, Wei Y, Song Y, Chen P, Zhou Z, Zhang N, He Q, Liu L (2021). Association of serum choline levels and all-cause mortality risk in adults with hypertension: a nested case-control study. Nutr metabolism.

[11] Danne O, Möckel M (2010). Choline in acute coronary syndrome: an emerging biomarker with implications for the integrated assessment of plaque vulnerability. Expert Rev Mol Diagn.

[12] Danne O, Möckel M, Lueders C, Mügge C, Zschunke GA, Lufft H, Müller C, Frei U (2003). Prognostic implications of elevated whole blood choline levels in acute coronary syndromes. Am J Cardiol.

[13] Pulido T, Adzerikho I, Channick RN, Delcroix M, Galiè N, Ghofrani HA, Jansa P, Jing ZC, Le Brun FO, Mehta S (2013). Macitentan and morbidity and mortality in pulmonary arterial hypertension. N Engl J Med.

[14] Galiè N, Humbert M, Vachiery JL, Gibbs S, Lang I, Torbicki A, Simonneau G, Peacock A, Vonk Noordegraaf A, Beghetti M (2016). 2015 ESC/ERS guidelines for the diagnosis and treatment of pulmonary hypertension: the Joint Task Force for the diagnosis and treatment of pulmonary hypertension of the European Society of Cardiology (ESC) and the european respiratory society (ERS): endorsed by: Association for European Paediatric and congenital cardiology (AEPC), International Society for Heart and Lung Transplantation (ISHLT). Eur Heart J.

[15] Konstantinova SV, Tell GS, Vollset SE, Nygård O, Bleie Ø, Ueland PM (2008). Divergent associations of plasma choline and betaine with components of metabolic syndrome in middle age and elderly men and women. J Nutr.

[16] Fischer LM, daCosta KA, Kwock L, Stewart PW, Lu TS, Stabler SP, Allen RH, Zeisel SH (2007). Sex and menopausal status influence human dietary requirements for the nutrient choline. Am J Clin Nutr.

[17] Haubrich DR, Wang PF, Wedeking PW (1975). Distribution and metabolism of intravenously administered choline[methyl- 3-H] and synthesis in vivo of acetylcholine in various tissues of guinea pigs. J Pharmacol Exp Ther.

[18] Bligh J (1953). The role of the liver and the kidneys in the maintenance of the level of free choline in plasma. J Physiol.

[19] Thenappan T, Ormiston ML, Ryan JJ, Archer SL (2018). Pulmonary arterial hypertension: pathogenesis and clinical management. BMJ (Clinical research ed).

[20] Gardiner JE, Gwee MC (1974). The distribution in the rabbit of choline administered by injection or infusion. J Physiol.

[21] LEE DCP. : FACTORS CONTROLLING THE CHOLINE LEVEL IN THE CIRCULATION. 1971.

[22] Riou M, Pizzimenti M, Enache I, Charloux A, Canuet M, Andres E, Talha S, Meyer A, Geny B. Skeletal and respiratory muscle dysfunctions in pulmonary arterial hypertension. J Clin Med 2020, 9(2). 10.3390/jcm9020410 PMC7073630 32028638

[23] Bekfani T, Bekhite Elsaied M, Derlien S, Nisser J, Westermann M, Nietzsche S, Hamadanchi A, Fröb E, Westphal J, Haase D (2020). Skeletal muscle function, structure, and metabolism in patients with heart failure with reduced ejection fraction and heart failure with preserved ejection fraction. Circ Heart Fail.

[24] Jaitovich A, Barreiro E (2018). Skeletal muscle dysfunction in Chronic Obstructive Pulmonary Disease. What we know and can do for our patients. Am J Respir Crit Care Med.

[25] Solberg R, Kuligowski J, Pankratov L, Escobar J, Quintás G, Lliso I, Sánchez-Illana Á, Saugstad OD, Vento M (2016). Changes of the plasma metabolome of newly born piglets subjected to postnatal hypoxia and resuscitation with air. Pediatr Res.

[26] Scremin OU, Jenden DJ (1993). Acetylcholine turnover and release: the influence of energy metabolism and systemic choline availability. Prog Brain Res.

[27] Nilsson Ã, Duan RD (2019). Pancreatic and mucosal enzymes in choline phospholipid digestion. Am J Physiol Gastrointest liver Physiol.

[28] Zhu W, Gregory JC, Org E, Buffa JA, Gupta N, Wang Z, Li L, Fu X, Wu Y, Mehrabian M (2016). Gut Microbial Metabolite TMAO enhances platelet hyperreactivity and thrombosis risk. Cell.

[29] Organ CL, Otsuka H, Bhushan S, Wang Z, Bradley J, Trivedi R, Polhemus DJ, Tang WH, Wu Y, Hazen SL (2016). Choline Diet and its gut microbe-derived metabolite, trimethylamine N-Oxide, exacerbate pressure Overload-Induced Heart failure. Circ Heart Fail.

[30] Seldin MM, Meng Y, Qi H, Zhu W, Wang Z, Hazen SL, Lusis AJ, Shih DM. Trimethylamine N-Oxide promotes vascular inflammation through signaling of Mitogen-Activated protein kinase and nuclear Factor-κB. J Am Heart Association 2016, 5(2). 10.1161/JAHA.115.002767 PMC4802459 26903003

[31] Huang Y, Lin F, Tang R, Bao C, Zhou Q, Ye K, Shen Y, Liu C, Hong C, Yang K (2022). Gut microbial metabolite trimethylamine N-Oxide aggravates pulmonary hypertension. Am J Respir Cell Mol Biol.

[32] Wang Z, Klipfell E, Bennett BJ, Koeth R, Levison BS, Dugar B, Feldstein AE, Britt EB, Fu X, Chung YM (2011). Gut flora metabolism of phosphatidylcholine promotes cardiovascular disease. Nature.

[33] Sanli C, Oguz D, Olgunturk R, Tunaoglu FS, Kula S, Pasaoglu H, Gulbahar O, Cevik A (2012). Elevated homocysteine and asymmetric dimethyl arginine levels in pulmonary hypertension associated with congenital heart disease. Pediatr Cardiol.

[34] Sun L, Sun S, Li Y, Pan W, Xie Y, Wang S, Zhang Z (2014). Potential biomarkers predicting risk of pulmonary hypertension in congenital heart disease: the role of homocysteine and hydrogen sulfide. Chin Med J.

