# Supplementary material for: Circulating choline levels are associated with prognoses in patients with pulmonary hypertension: a cohort study
Source: BMC Pulm Med. 2023 Sep 10;23:313. doi: 10.1186/s12890-023-02547-9 (PMC10493021; doi:10.1186/s12890-023-02547-9)
Supplement: Supplementary file 1 — Supplementary Material 1 [file 12890_2023_2547_MOESM1_ESM.docx]

**Supplementary Table 1. Correlations between choline and clinical variables in total PH patients**

| **Variables** | ***r*** | ***P* value** |
| --- | --- | --- |
| Age, years | **0.269** | **<0.001** |
| Sex | **-0.287** | **<0.001** |
| BMI, kg/m^2^ | 0.096 | 0.113 |
| **Laboratories** |  |  |
| NT-proBNP, pg/ml (categorical variable) | **0.339** | **<0.001** |
| ALT, IU/L | **0.160** | **0.008** |
| AST, IU/L | **0.186** | **0.002** |
| Creatinine, μM | **0.448** | **<0.001** |
| Total cholesterol, mM | -0.062 | 0.307 |
| Triglycerides, mM | 0.094 | 0.121 |
| Serum iron, μM | 0.071 | 0.247 |
| **Exercise capacity** |  |  |
| PeakVO_2_, mL/min/kg | **-0.239** | **<0.001** |
| VO_2_% | **-0.263** | **<0.001** |
| VCO_2_% | **-0.206** | **0.001** |
| 6MWD, m | **-0.171** | **0.013** |
| **Hemodynamics** |  |  |
| mRAP, mmHg | 0.057 | 0.410 |
| RVDP, mmHg | **0.143** | **0.039** |
| mPAP, mmHg | 0.005 | 0.942 |
| Cardiac output index, L/min*m^2^ | **-0.290** | **<0.001** |

PH: pulmonary hypertension; BMI: body mass index; WHO FC: world health organization function class; NT-proBNP: N-terminal pro-brain natriuretic peptide; ALT: alanine aminotransferase; AST: aspartate aminotransferase; 6MWT: 6-minute walk distance; mRAP: mean right atrial pressure; RVDP: [right ventricular diastolic pressure](http://www.baidu.com/link?url=ELtlANUndOsjtSqis0QRLCnseYJyGaayLWEh8tiO-Wg7CBTCz5O1zzrW8lqVESJd); mPAP: mean pulmonary atrial pressure.
