# Supplementary material for: Circulating choline levels are associated with prognoses in patients with pulmonary hypertension: a cohort study
Source: BMC Pulm Med. 2023 Sep 10;23:313. doi: 10.1186/s12890-023-02547-9 (PMC10493021; doi:10.1186/s12890-023-02547-9)
Supplement: Supplementary file 2 — Supplementary Material 2 [file 12890_2023_2547_MOESM2_ESM.docx]

**Supplementary Table 2. The associations between plasma choline and WHO-FC, NT-proBNP, and cardiac output index after adjusting for confounders in total PH patients**

| **Logistics analysis** | **OR** | **95%CI** | ***P*** |
| --- | --- | --- | --- |
| **Model 1: WHO-FC** |  |  |  |
| Unadjusted | 2.277 | 1.379-3.759 | **0.001** |
| Adjusted ^a^ | 3.967 | 1.444-10.901 | **0.008** |
| Adjusted ^b^ | 2.390 | 1.435-3.982 | **0.001** |
| **Model 2: NT-proBNP (categorical variable)** |  |  |  |
| Unadjusted | 2.861 | 1.721-4.755 | **<0.001** |
| Adjusted ^c^ | 3.275 | 1.322-8.110 | **0.010** |
| Adjusted ^b^ | 2.835 | 1.688-4.762 | **<0.001** |
| **Linear regression analysis** | **Beta** | **95%CI** | ***P*** |
| **Model 3: Cardiac output index (continuous variable)** |  |  |  |
| Unadjusted | -0.228 | -0.702 - -0.182 | **0.001** |
| Adjusted ^d^ | -0.187 | -0.706 - -0.027 | **0.035** |
| Adjusted ^b^ | -0.236 | -0.724 - -0.191 | **0.001** |

Plasma choline concentration was put into the model as a categorical variable. The continuous variable of choline and NT-proBNP were converted into a categorical variable with a boundary of 12.6 μM and 300pg/ml.

^a^ Adjusted for age, sex, NT-proBNP (categorical variable), ALT, AST, creatinine, VO_2_%, VCO_2_%, PeakVO_2_, and 6MWD;

^b^ Adjusted for hypertension, coronary heart disease, and diabetes;

^c^ Adjusted for age, sex, WHO-FC (categorical variable), ALT, AST, creatinine, VO_2_%, VCO_2_%, PeakVO_2_, and 6MWD;

^d^ Adjusted for age, sex, WHO-FC (categorical variable), NT-proBNP (categorical variable), creatinine, PeakVO_2_, and 6MWD.

PH: pulmonary hypertension; WHO FC: world health organization function class; NT-proBNP: N-terminal pro-brain natriuretic peptide; ALT: alanine aminotransferase; AST: aspartate aminotransferase; 6MWD: 6-minute walk distance.
