# Supplementary material for: Circulating choline levels are associated with prognoses in patients with pulmonary hypertension: a cohort study
Source: BMC Pulm Med. 2023 Sep 10;23:313. doi: 10.1186/s12890-023-02547-9 (PMC10493021; doi:10.1186/s12890-023-02547-9)
Supplement: Supplementary file 3 — Supplementary Material 3 [file 12890_2023_2547_MOESM3_ESM.docx]

**Supplementary Table 3. Univariate Cox regression analysis between choline and clinical variables in total PH patients**

| **Variable** | **HR** | **95% CI** | ***P*** |
| --- | --- | --- | --- |
| Age, year | 1.013 | 1.000-1.027 | **0.049** |
| Sex | 0.722 | 0.473-1.103 | 0.132 |
| BMI, kg/m^2^ | 0.991 | 0.943-1.041 | 0.725 |
| WHO-FC | 1.985 | 1.308-3.013 | **0.001** |
| Choline, μM (categorical variable) | 2.069 | 1.339-3.199 | **0.001** |
| NT-proBNP, pg/mL (categorical variable) | 1.897 | 1.180-3.050 | **0.008** |
| ALT, IU/L | 0.998 | 0.985-1.010 | 0.725 |
| AST, IU/L | 0.999 | 0.983-1.016 | 0.948 |
| Creatinine, μM | 1.002 | 1.000-1.005 | **0.021** |
| Total cholesterol, mM | 0.873 | 0.732-1.041 | 0.130 |
| Triglycerides, mM | 0.909 | 0.679-1.217 | 0.521 |
| Serum iron, μM | 0.990 | 0.963-1.017 | 0.460 |
| PeakVO_2_, mL/min/kg | 0.866 | 0.855-0.932 | **<0.001** |
| VO2% | 0.525 | 0.258-1.069 | 0.076 |
| VCO2% | 0.666 | 0.324-1.367 | 0.268 |
| 6MWD, m | 0.997 | 0.994-1.000 | **0.022** |
| mRAP, mmHg | 1.045 | 0.984-1.109 | 0.150 |
| RVDP, mmHg | 1.029 | 0.997-1.063 | 0.078 |
| mPAP, mmHg | 0.993 | 0.978-1.008 | 0.335 |
| Cardiac index, L/min*m^2^ | 0.782 | 0.603-1.015 | 0.065 |
| **Disease type** |  |  | 0.052 |
| PAH/Other | 0.581 | 0.284-1.185 | 0.135 |
| CTEPH/Other | 0.981 | 0.464-2.075 | 0.960 |
| **Comorbidities** |  |  |  |
| Hypertension | 1.068 | 0.621-1.837 | 0.811 |
| Coronary heart disease | 1.797 | 0.926-3.487 | 0.083 |
| Diabetes | 1.600 | 0.802-3.191 | 0.182 |

PH: pulmonary hypertension; BMI: body mass index; WHO FC: world health organization function class; NT-proBNP: N-terminal pro-brain natriuretic peptide; ALT: alanine aminotransferase; AST: aspartate aminotransferase; 6MWD: 6-minute walk distance; mRAP: mean right atrial pressure; RVDP: [right ventricular diastolic pressure](http://www.baidu.com/link?url=ELtlANUndOsjtSqis0QRLCnseYJyGaayLWEh8tiO-Wg7CBTCz5O1zzrW8lqVESJd); mPAP: mean pulmonary atrial pressure; PAH: pulmonary arterial hypertension; CTEPH: chronic thromboembolic pulmonary hypertension.
