# Supplementary material for: Circulating choline levels are associated with prognoses in patients with pulmonary hypertension: a cohort study
Source: BMC Pulm Med. 2023 Sep 10;23:313. doi: 10.1186/s12890-023-02547-9 (PMC10493021; doi:10.1186/s12890-023-02547-9)
Supplement: Supplementary file 4 — Supplementary Material 4 [file 12890_2023_2547_MOESM4_ESM.docx]

**Supplementary Table 4. Baseline characteristics in different subgroups stratified by circulating choline levels**

| **Variables** | **IPAH/HPAH patients**  **N=56** | | **CHD-PAH patients**  **N=101** | | **CTEPH patients**  **N=78** | |
| --- | --- | --- | --- | --- | --- | --- |
|  | **High choline**  **N=23** | **Low choline**  **N=33** | **High choline**  **N=43** | **Low choline**  **N=58** | **High choline**  **N=48** | **Low choline**  **N=30** |
| Age, years | 36.9±11.8 | 34.4±11.4 | 35.4±13.4 | 33.9±13.2 | 60.0±12.7**^#^** | 54.0±12.9 |
| Female sex, n (%) | 12 (52.2)**^#^** | 28 (84.8) | 24 (55.8)**^#^** | 50 (86.2) | 22 (45.8) | 14 (46.7) |
| BMI, kg/m^2^ | 22.6±3.2 | 22.6±3.8 | 21.1±3.0 | 20.4±5.0 | 23.6±3.6 | 24.5±3.2 |
| **WHO-FC, n (%)** |  |  |  |  |  |  |
| I-II | 7 (30.4)* | 23 (69.7) | 25 (58.1)* | 44 (75.9) | 28 (58.3)**^#^** | 22 (73.3) |
| III-IV | 16 (69.6)* | 10 (30.3) | 18 (41.9)* | 14 (24.1) | 20 (41.7)**^#^** | 8 (26.7) |
| **Laboratories** |  |  |  |  |  |  |
| Choline, μM | 17.7 (15.2, 22.3)* | 10.0 (8.1, 11.3) | 15.2 (13.9, 17.9)* | 10.0 (8.5, 11.6) | 17.0 (14.5, 20.4)* | 10.4 (9.6, 11.4) |
| NT-proBNP, pg/ml | 1448.0 (659.8, 2980.0)* | 580.6 (273.6, 1404.0) | 526.5 (167.8, 1895.0)* | 288.2 (136.0, 545.5) | 841.4 (152.5, 2177.8)* | 191.0 (78.1, 576.8) |
| ALT, IU/L | 26.0 (18.0, 39.0)**^#^** | 16.0 (11.5, 27.0) | 15.0 (9.0, 24.0) | 15.0 (9.0, 20.0) | 18.0 (11.3, 23.0) | 18.5 (15.0, 29.3) |
| AST, IU/L | 32.0 (26.0, 43.0) | 28.0 (24.0, 34.0) | 25.0 (21.0, 28.0) | 22.0 (20.0, 30.0) | 28.5 (22.3, 34.0) | 27.5 (22.8, 35.3) |
| Creatinine, μM | 90.0 (72.0, 97.7)**^#^** | 69.6 (63.1, 81.3) | 79.0 (71.2, 95.0)* | 70.0 (69.9, 76.2) | 89.5 (76.3, 98.0)**^#^** | 78.1 (66.9, 85.0) |
| Total cholesterol, mM | 4.2±0.8 | 4.0±1.1 | 4.0±1.1 | 4.1±0.9 | 4.3±1.1 | 4.7±1.3 |
| Triglycerides, mM | 1.4±0.5 | 1.2±0.6 | 1.2±0.5 | 1.2±1.5 | 1.2±0.6 | 1.4±0.6 |
| Serum iron, μM | 16.3±8.1 | 15.0±7.0 | 16.5±9.2 | 14.3±8.9 | 15.7±6.6 | 16.4±6.0 |
| **Exercise capacity** |  |  |  |  |  |  |
| PeakVO_2_, mL/min/kg | 13.7±2.7 | 14.7±4.3 | 13.4±4.5 | 15.0±3.7 | 13.0±3.9**^#^** | 15.9±3.3 |
| VO_2_% | 1.4±0.2 | 1.6±0.4 | 1.4±0.4**^#^** | 1.7±0.3 | 1.3±0.3 | 1.4±0.2 |
| VCO_2_% | 1.4±0.2 | 1.4±0.3 | 1.3±0.4**^#^** | 1.6±0.4 | 1.3±0.3 | 1.3±0.2 |
| 6MWD, m | 392.6±105.4 | 438.3±92.9 | 398.9±108.7 | 427.6±87.6 | 407.1±100.8 | 451.0±78.3 |
| **Hemodynamics** |  |  |  |  |  |  |
| mRAP, mmHg | 5.8±4.5 | 6.1±4.0 | 7.4±5.4**^#^** | 5.1±3.1 | 6.9±3.8 | 8.2±3.9 |
| RVDP, mmHg | -1.5 (-4.5, 5.0) | -2.5 (-6.5, 2.5) | 2.0 (-5.0, 6.0)**^#^** | -4.0 (-6.3, 1.3) | -1.0 (-4.5, 2.5) | 0.0 (-4.0, 4.0) |
| mPAP, mmHg | 64.8±12.1 | 59.0±11.3 | 70.4±20.5 | 65.8±20.3 | 45.8±10.0 | 45.7±11.3 |
| Cardiac index, L/min*m^2^ | 2.6±0.7 | 2.8±0.8 | 3.2±1.1**^#^** | 3.7±1.1 | 2.8±0.7**^#^** | 3.3±0.7 |
| **Treatment** |  |  |  |  |  |  |
| ERA | 17 (73.9) | 23 (69.7) | 28 (65.1) | 42 (72.4) | 5 (10.4) | 3 (10.0) |
| NO pathway | 21 (91.3) | 24 (72.7) | 8 (18.6) | 10 (17.2) | 37 (77.1) | 23 (76.7) |
| Prostacyclin analogues | 8 (34.8) | 11 (33.3) | 35 (81.4) | 46 (79.3) | 1 (2.1) | 0 (0.0) |

PH patients were stratified into low and high choline groups by 50^th^ percentile of plasma choline levels (12.6 μM). IPAH/HPAH: idiopathic/hereditary pulmonary arterial hypertension; CHD-PAH: pulmonary arterial hypertension associated with congenital heart disease; CTEPH: chronic thromboembolic pulmonary hypertension; BMI: body mass index; WHO FC: world health organization function class; NT-proBNP: N-terminal pro-brain natriuretic peptide; ALT: alanine aminotransferase; AST: aspartate aminotransferase; 6MWD: 6-minute walk distance; mRAP: mean right atrial pressure; RVDP: [right ventricular diastolic pressure](http://www.baidu.com/link?url=ELtlANUndOsjtSqis0QRLCnseYJyGaayLWEh8tiO-Wg7CBTCz5O1zzrW8lqVESJd); mPAP: mean pulmonary atrial pressure; ERA: endothelium-Receptor Antagonist; NO: nitric oxide.
