# Supplementary material for: Circulating choline levels are associated with prognoses in patients with pulmonary hypertension: a cohort study
Source: BMC Pulm Med. 2023 Sep 10;23:313. doi: 10.1186/s12890-023-02547-9 (PMC10493021; doi:10.1186/s12890-023-02547-9)
Supplement: Supplementary file 5 — Supplementary Material 5 [file 12890_2023_2547_MOESM5_ESM.docx]

**Supplementary Table 5. Univariate Cox regression analysis between choline and clinical variables in PAH subgroup patients**

| **Variable** | **HR** | **95% CI** | ***P*** |
| --- | --- | --- | --- |
| Age, years | 0.989 | 0.968-1.011 | 0.336 |
| Sex | 0.610 | 0.342-1.088 | 0.094 |
| BMI, kg/m^2^ | 0.990 | 0.937-1.047 | 0.734 |
| WHO-FC | 2.298 | 1.324-3.998 | **0.003** |
| Choline, μM (categorical variable) | 2.029 | 1.165-3.534 | **0.012** |
| NT-proBNP, pg/mL (categorical variable) | 0.774 | 0.445-1.346 | 0.364 |
| ALT, IU/L | 1.008 | 0.991-1.024 | 0.367 |
| AST, IU/L | 1.007 | 0.986-1.030 | 0.502 |
| Creatinine, μM | 1.005 | 0.991-1.019 | 0.474 |
| Total cholesterol, mM | 1.086 | 0.836-1.410 | 0.536 |
| Triglycerides, mM | 1.233 | 1.012-1.503 | **0.038** |
| Serum iron, μM | 1.008 | 0.970-1.048 | 0.668 |
| PeakVO_2_, mL/min/kg | 1.033 | 0.964-1.108 | 0.358 |
| VO_2_% | 1.400 | 0.583-3.362 | 0.451 |
| VCO_2_% | 1.730 | 0.718-4.171 | 0.222 |
| 6MWD, m | 0.997 | 0.993-1.000 | **0.040** |
| mRAP, mmHg | 0.990 | 0.914-1.073 | 0.806 |
| RVDP, mmHg | 1.011 | 0.971-1.053 | 0.587 |
| mPAP, mmHg | 1.003 | 0.984-1.023 | 0.729 |
| Cardiac output index, L/min*m^2^ | 0.810 | 0.550-1.193 | 0.287 |
| **Comorbidities** |  |  |  |
| Hypertension | 0.566 | 0.203-1.574 | 0.275 |
| Coronary heart disease | 0.888 | 0.214-3.683 | 0.871 |
| Diabetes | 1.051 | 0.326-3.386 | 0.934 |

PAH: pulmonary arterial hypertension; BMI: body mass index; WHO FC: world health organization function class; NT-proBNP: N-terminal pro-brain natriuretic peptide; ALT: alanine aminotransferase; AST: aspartate aminotransferase; 6MWD: 6-minute walk distance; mRAP: mean right atrial pressure; RVDP: [right ventricular diastolic pressure](http://www.baidu.com/link?url=ELtlANUndOsjtSqis0QRLCnseYJyGaayLWEh8tiO-Wg7CBTCz5O1zzrW8lqVESJd); mPAP: mean pulmonary atrial pressure.
